# Structural mechanism of 26S proteasome regulation and its pharmacological modulation

**DOI:** 10.1016/j.jbc.2026.113256

**Published:** 2026-06-16

**Authors:** Kai Pei, Lihong Zhao, Jiao Hu, Youdong Mao

**Affiliations:** 1 Peking-Tsinghua Joint Center for Life Sciences, Peking University, Beijing, China; 2 State Key Laboratory for Artificial Microstructures and Mesoscopic Physics, School of Physics, Peking University, Beijing, China; 3 National Biomedical Imaging Center, Peking University, Beijing, China; 4 Center for Quantitative Biology, Peking University, Beijing, China; 5 School of Chemical Biology and Biotechnology, Shenzhen Graduate School, Peking University, Shenzhen, China

**Keywords:** 26S proteasome, AAA+ ATPase, cryo-EM, pharmacological modulation, proteostasis, homeostasis, protein quality control, allosteric regulation, proteasome inhibitor, targeted proteindegradation, molecular glue degrader, proteolysis-targeting chimera

## Abstract

The proteasome is a conserved proteolytic machinery that maintains protein quality control and regulates nearly every aspect of cellular activities. It is a clinically validated therapeutic target in cancer and holds great potential in therapeutic discovery for protein aggregation–associated disorders. Advances in structural biology have elucidated the architecture and conformational dynamics of the 26S proteasome, providing mechanistic insights into substrate recognition, unfolding, translocation, and proteolysis by the proteasome and pharmacological intervention. This review article summarizes current knowledge on the structural basis of substrate processing by the 26S proteasome and highlights emerging regulatory mechanisms mediated by enzymatic cofactors. We outline the molecular principles underlying current inhibitors and activators targeting various components of the proteasome, as well as targeted-degradation strategies directly involving the proteasome. We further discuss how new views of proteasome dynamics and allosteric regulation by protein cofactors and small molecules may shape the rational design of next-generation proteasome modulators with broadened therapeutic applications.

Protein degradation is essential for maintaining cellular homeostasis and regulating cell fate (1). In eukaryotic cells, two major systems mediate protein turnover: the lysosomal pathway, which degrades damaged organelles and protein aggregates, and the ubiquitin-proteasome system (UPS), which selectively eliminates short-lived, misfolded, and regulatory proteins to control processes such as cell cycle progression, DNA replication, DNA repair, and immune signaling (2). The proteasome plays a central role in protein quality control by removing oxidatively damaged and misfolded soluble proteins and regulating diverse intracellular signaling pathways (3, 4).

Human cells contain multiple forms of proteasome assemblies, including the 20S core particle (CP), the 26S/30S proteasome, and the immunoproteasome. In the immunoproteasome, the catalytic β-subunits, which contain the proteolytic active sites, are replaced by β1i (LMP2), β2i (MECL-1), and β5i (LMP7), generating peptides optimized for antigen presentation (5). The 26S proteasome, responsible for the majority of selective protein degradation, consists of a CP capped by one or two 19S regulatory particles (RPs), forming 26S (RP–CP) or 30S (RP_2_–CP) complexes (6). The CP is composed of four stacked heptameric rings arranged in an α_7_–β_7_–β_7_–α_7_ configuration. The outer α rings regulate substrate entry, whereas the inner β rings contain the proteolytic active sites. The RP, also known as PA700, comprises a lid and a base subcomplex. The lid contains multiple regulatory particle non-ATPase (RPN) subunits that recognize polyubiquitinated substrates, proteins modified with multiple ubiquitin molecules, and catalyze deubiquitylation, the removal of ubiquitin tags from substrate proteins. In contrast, the base contains six AAA+ ATPases (RPT1–RPT6) that drive substrate unfolding, CP gate opening, and translocation into the CP (7, 8).

The proteasome has emerged as a validated therapeutic target. Since 2003, several CP-targeting inhibitors have been approved for the treatment of multiple myeloma (MM) and related malignancies. Inhibitors targeting RP components have also been reported to suppress 26S proteasome activity (9, 10, 11). The CP contributes to the degradation of intrinsically disordered, oxidized, and misfolded proteins, which are commonly associated with neurodegenerative disorders, suggesting that pharmacological activation of the CP may provide therapeutic benefit (12). With the rapid expansion of high-resolution proteasome structures, a mechanistic understanding of proteasome regulation is increasingly enabling structure-guided modulation of proteasome activity. Moving forward, integrating structural insights with chemical biology and disease-specific targeting strategies may allow precise and context-dependent manipulation of proteasome function for next-generation therapeutic development.

## The RP

**Figure 1:**
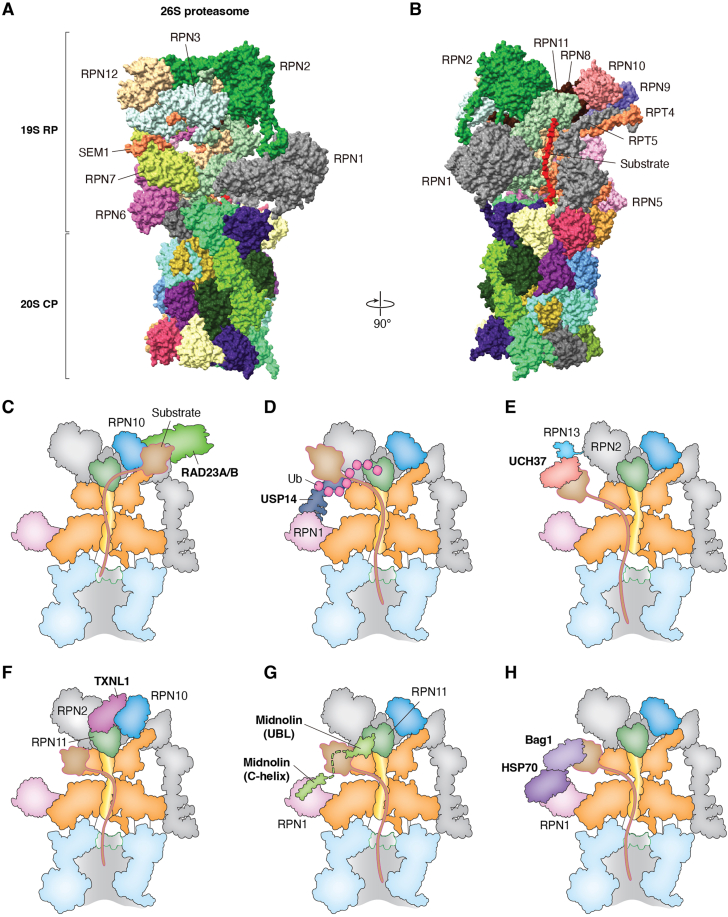
**Architecture of the 26S proteasome and schematic representation of proteasome-associated cofactors.***A* and *B*, cryo-EM structure of the human 26S proteasome shown in two orientations (approximately 90° rotation). The 19S regulatory particle (RP) and the 20S core particle (CP) are indicated. Individual subunits of the RP are color-coded and labeled, including RPN1, RPN2, RPN3, RPN5, RPN6, RPN7, RPN8, RPN9, RPN10, RPN11, RPN12, RPT4, RPT5, and SEM1. *C–H*, schematic representations of reported interactions between the RP and various cofactors. The RP subunits are shown in different colors. The labeled subunits indicate those that can interact with cofactors. *C*, RAD23A/RAD23B contains an N-terminal ubiquitin-like (UBL) domain and has been reported to associate with Rpn10. *D*, USP14 has been shown to bind RPN1 and influence ubiquitin chain processing at the proteasome. *E*, UCH37 interacts with RPN13 and contributes to ubiquitin chain remodeling at the proteasome. *F*, TXNL1 has been reported to associate with the RP, through RPN10 and RPN2. *G*, midnolin has been reported to associate with the proteasome, with evidence suggesting an interaction involving RPN1 and RPN11. *H*, the Bag1–Hsp70 complex associates with RPN1 through the ubiquitin-like (UBL) domain of Bag1. The *pink ball* represents Ub, and the *brown leafy structure* represents substrate. RP, regulatory particle.

The RP plays a critical role in the proteasomal degradation pathway by recognizing polyubiquitinated substrates, removing ubiquitin chains, and unfolding proteins for delivery to the CP (6, 13, 14). The lid subcomplex of RP is responsible for substrate recognition, while the base subcomplex is the engine driving substrate unfolding and translocation. The interaction between the lid and base is partly mediated by RPN2, which links to the N-terminal coiled-coil regions of RPT3 and RPT6, and by the tetratricopeptide repeat domains of RPN5, RPN6, and RPN7 that engage with the ATPase domains of RPT4, RPT3, and RPT6 (15, 16). The lid subassembly, formed by noncatalytic subunits (RPN3, RPN5, RPN6, RPN7, RPN8, RPN9, RPN12, and SEM1), serves as a scaffold that supports recognition of polyubiquitin chains, while the base subcomplex, composed of RPN1, RPN10, RPN11, RPN13, and the six ATPases (RPT1–RPT6) (Fig. 1, *A* and *B*), facilitates unfolding and translocation of substrates into the CP (17). The AAA-ATPase ring of the base generates mechanical energy *via* ATP hydrolysis to unfold substrates and drive their entry into the CP gate. This process is essential for the selective degradation of damaged, misfolded, or regulatory proteins.

RPN1, RPN10 and RPN13 function as ubiquitin receptors, recognizing and binding primarily K48-linked ubiquitin chains and ubiquitin-like (UBL) domain-containing substrates. RPN11, a Zn^2+^-dependent deubiquitinase, removes ubiquitin chains from substrates (18). The six AAA-ATPases form a hexameric ring, with each subunit interacting with its neighbors *via* coiled-coil structures, creating a central channel (∼10 Å in diameter) through which substrates are translocated and unfolded during ATP hydrolysis (19). The mechanical energy from ATP hydrolysis drives substrate unfolding and gate opening, enabling delivery to the CP for degradation (20). The C-terminal tails of RPT subunits (except RPT4) insert into the α-ring of the CP, promoting gate opening and facilitating substrate engagement.

The RP also interacts with various cofactors that modulate proteasome activity. Shuttle factors such as RAD23A and RAD23B (HR23A and HR23B) in humans and their yeast ortholog Rad23 bind to RPN10 *via* their UBL domains (Fig. 1*C*), promoting substrate degradation (21, 22, 23). Deubiquitylating enzymes (DUBs) such as human USP14 (and its yeast ortholog Ubp6) bind to RPN1 (Fig. 1*D*) and dynamically associate with the ATPase subunits RPT1 and RPT2, thereby functionally uncoupling ATP hydrolysis from RPN11-mediated substrate deubiquitylation. In addition, association with the 26S proteasome markedly enhances the DUB activity of Ubp6/USP14 (24, 25). UCH37 similarly removes ubiquitin chains from substrates; it is recruited to the proteasome by RPN13 *via* its C-terminal KEKE motif (Fig. 1*E*), which also activates UCH37 activity (26, 27). E3 ligases, including UBE3C, E6AP, and Parkin, can interact with the RP and modulate 26S proteasome activity (28, 29, 30, 31).

Beyond DUBs and E3s, various proteasome-interacting cofactor proteins serve as surrogates for substrate recruitment for specific proteolytic needs. For example, TXNL1, a thioredoxin-fold protein, binds on top of RPN11 and contacts RPN2 and the VWA domain of RPN10 through its PITH domain, assisting substrate degradation and potentially reducing disulfide bonds with its N-terminal thioredoxin domain (Fig. 1*F*) (32, 33). Midnolin has recently been identified as a proteasome-interacting cofactor that mediates ubiquitin-independent degradation by interacting with RPN1 *via* its C-helix and with RPN11 *via* its N-terminal UBL domain (Fig. 1*G*) (34, 35, 36, 37). A zinc-finger protein ZFAND5 promotes protein degradation during muscle atrophy, by interacting with RPT1, RPT5, and RPN1 and facilitating ubiquitylated substrate recruitment (38, 39). Hsp70 family chaperones hydrolyze ATP to refold misfolded proteins and prevent their aggregation, thereby protecting cells from proteotoxic damage (40). Under stress conditions, the 26S proteasome cooperates with molecular chaperones to eliminate unfolded or damaged proteins. The Hsp70 co-chaperone Bag1 interacts with RPN1 (Fig. 1*H*) through its UBL domain and delivers Hsp70-bound substrates to the 26S proteasome, promoting substrate engagement and facilitating CP gate opening by modulating ATPase conformational dynamics (41).

Beyond ubiquitylation, FAT10ylation, a posttranslational modification involving conjugation of the UBL modifier FAT10, serves as a distinct signal for proteasomal degradation, with the UBL and ubiquitin-associated domain-containing protein NEDD8 ultimate buster-1 long mediating the recruitment of FAT10-modified substrates to the 26S proteasome (42, 43). Cryo-EM studies reveal that FAT10 comprises two tandem UBL domains, and that NUB1 engages the RPN1 T2 site to facilitate delivery of FAT10-conjugated substrates to the proteasome (44).

## The CP

**Figure 2:**
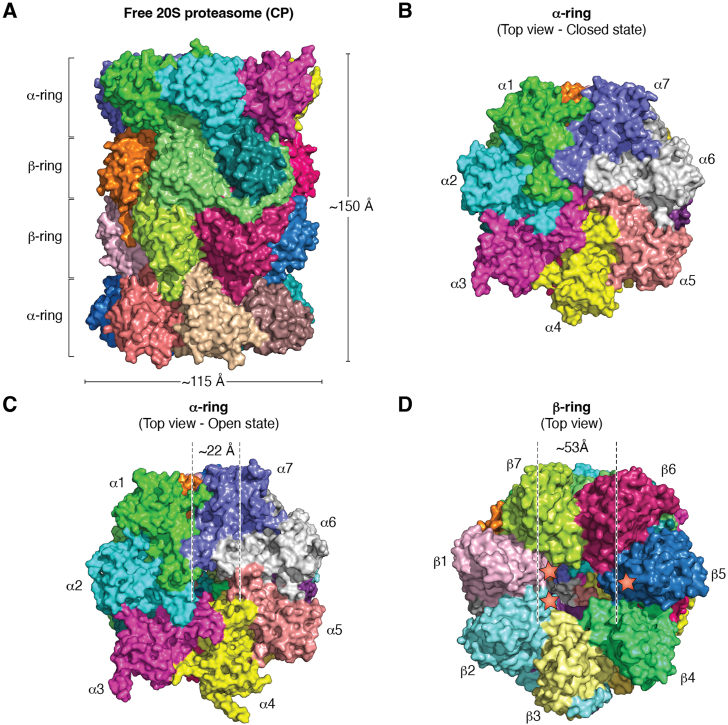
**Structures of the human CP.***A*, side view of the human CP (PDB: 6RGQ). The four stacked heptameric rings are organized in an α–β–β–α arrangement and are shown in distinct colors. The overall height of the CP is approximately 150 Å, with a diameter of approximately 115 Å. *B*, top view of the α-ring in the closed conformation (PDB: 4R3O). The seven α subunits (α1–α7) form a heptameric ring that defines the entrance to the proteolytic chamber. The central gate is shown in the closed state. *C*, top view of the α-ring in an open conformation (PDB: 9CE7). In this state, rearrangement of the N-terminal tails of the α subunits results in widening of the central pore, with a diameter of ∼22 Å. *D*, top view of the β-ring (PDB: 6MSJ). The seven β subunits (β1–β7) form the inner proteolytic chamber. The internal diameter of the β-ring is ∼53 Å. *Red stars* indicate the positions of the catalytically active β subunits. CP, core particle; PDB, Protein Data Bank.

The CP is a ∼700 kDa multi-subunit complex composed of 28 subunits, each ranging from 20 to 35 kDa (45). The CP adopts a barrel-shaped, four-ring architecture arranged in an α–β–β–α configuration (α7–β7–β7–α7), exhibiting dimensions of approximately 150 × 115 Å (Fig. 2*A*). In the substrate-free state, the α-ring gate remains closed, whereas substrate engagement induces gate opening to a diameter of ∼22 Å, thereby permitting substrate entry into the proteolytic chamber (Fig. 2, *B* and *C*) (19, 46). The α1–α7 subunits lack catalytic activity and form a regulatory ring that controls substrate entry. Structural studies of the 26S proteasomes show that adjacent α-subunits form seven α-pockets on the surface of α-ring around the CP gate, five of which bind the C-terminal HbYX motif of RPT ATPases, thereby triggering CP gate opening (47, 48). In mammalian cells, several CP activators, including PA700 (19S), PA28 (11S), and PA200, regulate CP gate opening through interactions with α-pockets (49). Synthetic HbYX motif peptides can also bind α-pockets to stimulate gate opening and enhance 20S proteolysis (50). Notably, PI31 contains an HbYX motif but possesses an intrinsically disordered C-terminal region that directly interacts with catalytic sites to inhibit CP activity without itself being degraded (51, 52, 53).

Beneath each α-ring lies a β-ring composed of seven distinct β subunits (β1–β7) surrounding the proteolytic channel, with an internal channel of ∼53 Å (Fig. 2*D*). The catalytic subunits β1, β2, and β5 mediate substrate hydrolysis. Although they share similar structural arrangements, they exhibit distinct proteolytic activities: β1 displays caspase-like activity, β2 exhibits trypsin-like activity, and β5 confers chymotrypsin-like activity, collectively enabling the degradation of unfolded substrates into peptide fragments. β1, β2, and β5 belong to the N-terminal nucleophile hydrolase family (54). Like other N-terminal nucleophile hydrolases, they share a conserved αββα fold despite limited sequence homology (∼30%) (55). In the catalytic mechanism, the N-terminal Thr1 residue acts as the nucleophile. The γ-hydroxyl group of Thr1 attacks the carbonyl carbon of the substrate peptide bond, forming a covalent acyl-enzyme intermediate. Subsequent hydrolysis releases the cleaved peptide fragment and regenerates the active enzyme, thereby completing peptide bond cleavage (56, 57, 58). In contrast, β3, β4, and β6 lack the essential N-terminal nucleophilic residue, and in β7, substitutions at conserved catalytic positions disrupt the active-site configuration, thereby abolishing proteolytic activity.

## Substrate processing cycle of the 26S proteasome

Owing to the intrinsic conformational flexibility of the RP, high-resolution structures of the substrate-free 26S proteasome were determined using cryo-EM instead of X-ray crystallography (16, 59, 60). Initial high-resolution cryo-EM structures of substrate-engaged 26S proteasomes revealed several distinct intermediate states during substrate binding and processing and provided systematic insights into the molecular mechanism underlying proteasomal substrate degradation (19, 61). The substrate-engaged proteasome adopts at least seven distinct conformational states, termed E_A1_, E_A2_, E_B_, E_C1_, E_C2_, E_D1_, and E_D2_. These states correspond to sequential stages of the functional cycle, including initial ubiquitin recognition, substrate insertion in the AAA-ATPase, initiation of substrate translocation, and substrate degradation. Notably, RPN11 functions as a gatekeeper at the entrance to the central channel of the AAA-ATPase ring, coordinating substrate deubiquitylation and translocation. In the E_B_ state, the insert-1 loop of RPN11 forms a β-hairpin that engages the C-terminal strand of ubiquitin while simultaneously interacting with the RPT5 N-loop (residues 99–119). This configuration, coupled with coordinated action of RPN11 and AAA-ATPase motor, has been interpreted as the onset of substrate translocation and RPN11-catalyzed deubiquitylation. The CP gate remains closed in the E_A_, E_B_, and E_C_ states, but becomes open in the E_D_ states, allowing substrate to enter the inner chamber of the CP, while ATP hydrolysis occurs sequentially around the AAA-ATPase ring throughout the entire functional cycle.

**Figure 3:**
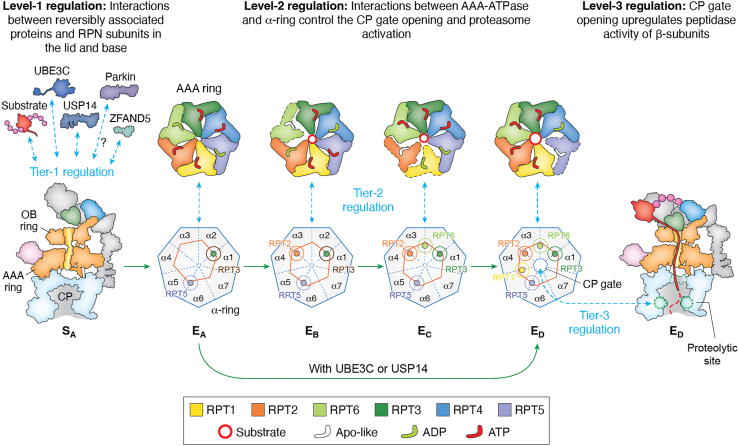
**Scheme for conformation-selective, hierarchical regulation of the proteasome by various proteasome-associated proteins.** The first level of regulation is *via* dynamic interactions between ubiquitin signals and an array of ubiquitin receptors, DUBs and other proteasome-interacting proteins. This is manifested as multifunctional roles of ubiquitin-binding sites in recognizing both ubiquitin signals and UBL proteins and in transmitting such protein–protein interactions into allosteric regulation of AAA-ATPase conformations that control the CP gate. The second level of proteasome regulation lies between the AAA-ATPase unfoldase and the rest of the proteasome. The AAA-ATPase motor bridges the architectural gap between the CP and RPN subunits including the lid subcomplex and the ubiquitin receptors in the base. Distinct dynamic modes of coordinated ATP hydrolysis appear to regulate the intermediate steps of substrate processing, in which initiation of substrate unfolding is allosterically coupled with CP gating. The last, innermost level of proteasome regulation is between the CP gate in the α-ring and the catalytic sites of the β-subunits. Opening of the CP gate facilitates substrate proteolysis by removing the physical barrier for substrate entry into the catalytic chamber. CP, core particle; DUB, deubiquitylating enzyme; RPN, regulatory particle non-ATPase; UBL, ubiquitin-like.

Furthermore, subsequent cryo-EM studies of the proteasome under various regulatory conditions resolved an extensive number of intermediate conformational states of the substrate-engaged proteasome, suggesting a hierarchy of regulation at the levels of the lid and ubiquitin receptors, the AAA-ATPase motor and the CP gate (Fig. 3) (32, 33, 37, 62, 63, 64). In a few reports, the substrate-engaged, CP gate-open, E_D_-compatible states of the proteasome were also referred to as processing states (32, 37). These studies collectively consolidate a picture of an asymmetric, hand-over-hand mechanism for the substrate translocation driven by pore loops of the six ATPase subunits arranged in a highly agile, dynamic spiral staircase architecture.

USP14 is one of the three DUBs associated with the proteasome. Unlike RPN11, which is an integral DUB subunit of the proteasome, USP14 is reversibly recruited to the proteasome and suppresses the proteasome activity by early removal of ubiquitin chains from substrates. Paradoxically, USP14 alone can also stimulate the proteasomal AAA-ATPase activity. Ultimately, the effect of USP14 depends on the temporal sequence: if substrate commitment precedes USP14 activation, the enhanced ATPase activity promotes degradation, but if USP14 is activated prior to substrate engagement, it instead inhibits the proteasomal degradation (24). To understand how these distinct, paradoxical effects of USP14 are integrated on the proteasome, time-resolved cryo-EM was used to visualize the USP14-bound proteasome in the act of polyubiquitylated substrate degradation (24). By integrating with deep neural network-based 3D classification of cryo-EM data, 13 distinct conformational states of the USP14-bound proteasome were resolved, providing mechanistic insights into USP14-mediated regulation of substrate processing in a spatiotemporal framework (62). The N-terminal UBL domain of USP14 binds to the T2 site of RPN1, while its catalytic USP domain engages the OB and AAA-ATPase rings and is positioned in close proximity to RPN11 (16, 24). Dynamic USP14–ATPase interactions uncouple ATPase activity from RPN11-catalyzed deubiquitylation and impose three kinetic checkpoints on the proteasome during ubiquitin recognition, substrate translocation initiation, and ubiquitin chain recycling (24).

Recently, time-resolved cryo-EM was further applied to visualize the functional dynamics of UBE3C-associated proteasome in the course of the degradation of substrates with ultrastable folds that otherwise resist proteasomal degradation despite ubiquitylation (63). Remarkably, the N-terminal segment of UBE3C, which remains disordered outside of the proteasome, inserts into a pocket near the central helical bundle of the lid subcomplex, in a key-lock fashion. Several cryo-EM reconstructions capturing the snapshots of substrate-engaged proteasome bound to both UBE3C and USP14 reveal an intricate antagonism of UBE3C against USP14. Intriguingly, while USP14 association with the proteasome promotes the UBE3C recruitment to the proteasome (65), UBE3C deprives USP14 of the ubiquitin-binding priority, by creating an extreme shortcut for ubiquitin shuttling from UBE3C to ubiquitin receptors and RPN11, bypassing USP14 altogether and facilitating USP14 recycling from the proteasome. Moreover, UBE3C appears to promote the AAA-ATPase activity independent of its ligase activity. These concerted actions result in the reversal of USP14-mediated suppression of the proteasome activity toward heightened degradative processivity, which is required for forceful degradation of ultrastable substrates. Taken together, these expanded cryo-EM studies, particularly with time-resolved approaches, define a more complete atomic-level functional landscape of the proteasome under various regulatory conditions that closely recapitulate its complex physiological functions in cells.

## Inhibitors targeting the RP

**Figure 4:**
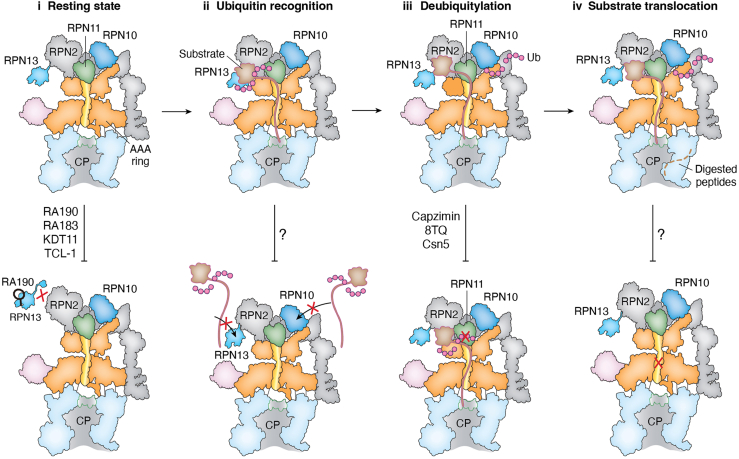
**Scheme of RP inhibitor interactions and their functional roles in substrate processing.** Schematic illustration of the RP functional cycle and the impact of inhibitors targeting distinct subunits. (*Top panel*) The substrate processing cycle: (i) In the resting state, the RP adopts an inactive conformation with the ATPase complex; (ii) upon polyubiquitinated substrate recognition, RPN10 and RPN13 UIM domains bind ubiquitin (Ub) chains, recruiting the substrate to the RP; (iii) RPN11-mediated deubiquitylation removes polyUb chains; and (iv) ATP hydrolysis drives substrate unfolding and translocation into the 20S core particle for degradation. (*Bottom panel*) Inhibitors targeting specific RP subunits disrupt distinct steps of substrate processing: RA190 covalently modifies RPN13, blocking substrate recruitment; RA183, KDT-11, and TCL-1 inhibit RPN13 function; Capzimin, 8TQ, and Csn5 inhibitors target RPN11, preventing deubiquitylation. X indicates the inhibited functional step. *Question marks* denote undefined inhibitory mechanisms. The *pink circles* represent ubiquitin chains, and the *brown leafy structure* represents the substrate. The *brown dotted line* in panel iv represents digested peptides derived from the substrate. The *black arrows* indicate substrate binding to the 26S proteasome subunit, while an “X” over the arrow denotes that this binding is blocked. RP, regulatory particle; RPN, regulatory particle non-ATPase; UIM, ubiquitin-interacting motif.

As discussed above, CP inhibitors have been developed for cancer therapy but are limited by dose-dependent toxicities, off-target effects, and acquired resistance. The RP represents an alternative therapeutic target for suppressing proteasome activity in cancer. In eukaryotic cells, the RP recognizes and processes ubiquitinated substrates through its distinct subunits, thereby providing multiple potential drug targets that may reduce off-target toxicity and resistance by complementing or substituting for CP-directed strategies (66). In principle, inhibition of the RP, similar to CP inhibition, can suppress proteasome activity in cancer cells, leading to substrate protein accumulation and triggering autophagic cell death (67, 68). Small molecules can disrupt ubiquitin recognition, ubiquitin chain removal, and ATP hydrolysis, thereby inhibiting proteasome function (Fig. 4) (19, 61).

RPN10 and RPN13 are ubiquitin receptors located at the top of the RP that recognize specific polyubiquitin chains (13). Human RPN10 contains two ubiquitin-interacting motifs (UIMs), whereas RPN13 contains one UIM capable of binding ubiquitin chains (69). Mutation of the RPN10 UIM domain is lethal in mice, whereas targeting RPN13 induces MM cell death and overcomes proteasome inhibitor resistance (70, 71). These findings suggest that RPN13 is a promising therapeutic target. Structural analyses of the RP indicate that the N-terminal pleckstrin-like receptor for ubiquitin (Pru) domain interacts with RPN2 and polyubiquitinated substrates (72). RA375, RA190, and RA183 covalently bind to Cys88 of RPN13, thereby inhibiting 26S proteasome activity. NMR studies demonstrate that RA190 binds to Cys88 of RPN13 near the RPN13–RPN2 interface, disrupting RPN13 docking to the proteasome (72). RA183 is a derivative of RA190 in which the m,p-chloro groups of bis-benzylidinepiperidone are replaced with p-nitro substituents and has shown activity against high-grade ovarian cancer, triple-negative breast cancer, and MM cells (66). Based on RA190, RA375 incorporates nitro ring substituents and a chloroacetamide warhead serving as a reactive chemical group that covalently binds target proteins, resulting in approximately tenfold greater activity against cancer cell lines than RA190 (73). KDT-11 is a peptoid—a peptide-like synthetic molecule—that noncovalently binds RPN13 without disrupting known protein–protein interactions and interacts with the β6/β7/β8 strands and α-helix of the RPN13 Pru domain (74, 75). TCL-1 is another noncovalent binder that interacts with the Pru domain and disrupts the RPN2–RPN13 interface (76).

Following polyubiquitin chain recruitment, the RP employs DUBs to remove ubiquitin chains from substrates (77). RPN11 is a member of the JAMM/MPN metalloprotease family and cleaves isopeptide bonds in ubiquitin–substrate conjugates (78). Within the 26S proteasome, RPN11 activity is coupled to substrate translocation and degradation in an ATP-dependent manner (79). Capzimin is an effective RPN11 inhibitor that binds tightly to the enzyme’s active site and inhibits its activity *via* a noncompetitive mechanism (79, 80). 8TQ is a coordination complex designed to bind the zinc ion at the catalytic site of RPN11, thereby inhibiting its activity (81). Thiolutin is a zinc chelator that inhibits RPN11 as well as other JAMM metalloproteases, including Csn5, AMSH, and BRCC36 (82). SOP6 and SOP11 belong to the epidithiodiketopiperazine class and inhibit RPN11 activity, although their mechanisms of action remain unclear (83).

More recently, a macrocyclic rapafucin compound, rapaprotin, was discovered to function as a 26S proteasome assembly inhibitor (84). Rapaprotin is a macrocyclic prodrug from a rapafucin library, the inactive precursor of which is converted into an active linearized form in cells that selectively induces apoptosis in MM and other hematologic cancer cells while sparing nonmalignant cells. A genome-wide CRISPR–Cas9 screen identified prolyl endopeptidase as essential for rapaprotin’s activity, leading to the discovery that prolyl endopeptidase converts the inactive cyclic compound into a linear metabolite, rapaprotin-L. Rapaprotin-L accumulates intracellularly, directly inhibits all three proteolytic β-subunits of the CP, and blocks ubiquitin–proteasome function, triggering accumulation of K48-linked polyubiquitinated proteins and unfolded protein response signaling. Time-resolved cryo-EM of human 26S proteasome treated with rapaprotin-L reveals stable intermediates lacking the lid and eventual dissociation of the RP, establishing a disassembly-based inhibitory mechanism. Rapaprotin synergizes strongly with bortezomib, carfilzomib, and ixazomib, and resensitizes bortezomib-resistant MM cells from patients, highlighting its potential as a first-in-class proteasome assembly inhibitor.

Together, these findings highlight the therapeutic potential of selectively targeting regulatory steps of the proteasome, either inhibiting its functional subunits or triggering its disassembly. By interfering with ubiquitin recognition, deubiquitylation, or ATP-driven substrate processing, RP-directed inhibitors may complement catalytic-site blockade and expand the scope of proteasome-targeted therapy. Further structural and mechanistic characterization of the RP will be critical for translating these strategies into clinically viable agents.

## Therapeutic targeting of the CP

Cancer cells depend on elevated proteasome activity to maintain proteostasis and sustain proliferation (85, 86). Proteasome inhibition results in accumulation of misfolded proteins and induction of apoptosis (87, 88, 89). In addition, inhibition of proteasomal degradation prevents IκB turnover and suppresses NF-κB–dependent transcription, contributing to tumor cell death (90). Proteasomes in malignant cells are generally more sensitive to inhibition than those in normal tissues (91).

**Figure 5:**
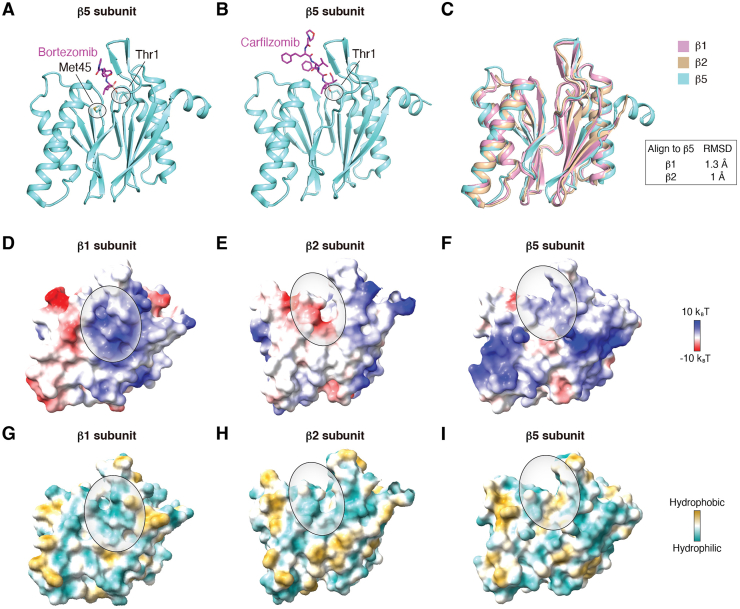
**Structural comparison of the proteasomal β1, β2, and β5 subunits.***A*, Atomic structure of the human β5 subunit in complex with bortezomib (PDB: 5LF3). Bortezomib is shown in *magenta sticks* within the catalytic pocket. *B*, Atomic structure of β5 bound to carfilzomib (PDB: 4R67). Carfilzomib is shown in *magenta sticks* occupying the active-site pocket. *C*, structural superposition of β1, β2, and β5. Subunits are colored as indicated. The overall folds are highly similar, with pairwise root-mean-square deviations (RMSDs) of approximately 1 Å. *D–F*, electrostatic surface representations of β1, β2, and β5, respectively. Surface potentials are displayed on the solvent-accessible surface, with *red* indicating negatively charged regions and *blue* indicating positively charged regions. The *circled areas* correspond to the catalytic pocket. *G*, *H*, and *I*, surface hydrophobicity analyses of β1, β2, and β5. Hydrophobic and hydrophilic regions are mapped onto the solvent-accessible surface as indicated by the color scale. The *circled regions* denote the substrate-binding pocket. For panels *C*–*I*, structures were derived from PDB 6RGQ and processed under identical parameters for structural alignment and surface calculations. Note: While functional protease sites are formed at the interface of neighboring β subunits, this schematic displays individual β1, β2, and β5 subunits to specifically highlight the unique architectures of their primary catalytic pockets for selective inhibitor design. PDB, Protein Data Bank.

Although the catalytic β1, β2, and β5 subunits of the 20S proteasome share a conserved N-terminal threonine–dependent hydrolytic mechanism (Fig. 5*C*), their substrate specificities are determined by distinct active-site architectures (92). Structural and physicochemical differences within the S1 specificity pockets, which accommodate the P1 side chains of substrates, generate subunit-specific microenvironments that govern peptide recognition and catalytic efficiency (93, 94, 95). Thr1 is positioned at the catalytic center adjacent to the S1 cavity, while residue 45, together with other pocket-lining residues, contributes to defining the size, depth, and electrostatic properties of the binding site. High-resolution structural analyses of the human CP further demonstrate how small molecules exploit these subunit-specific features to achieve selective engagement of individual catalytic centers (46, 56, 93, 96, 97). Structural analyses show that the S1 specificity pocket of β1 contains basic residues favoring acidic substrates, the β2 pocket is enriched in acidic residues accommodating basic side chains, and the β5 pocket forms a hydrophobic cavity that accommodates bulky aromatic residues typical of high-affinity inhibitors (95, 98) (Fig. 5, *D*–*I*). Thus, subunit-selective inhibition can be rationalized by differences in the local active-site environments within a conserved N-terminal threonine–based catalytic mechanism.

MG132 is a peptide aldehyde that preferentially inhibits the chymotrypsin-like activity of β5 (99). It acts as a reversible inhibitor in the low nanomolar range, while inhibiting β1 and β2 at higher concentrations (100). MG132 also inhibits calpains and cathepsins, leading to off-target effects (101, 102). Bortezomib, approved in 2003 for the treatment of MM and mantle cell lymphoma (103, 104), reversibly targets the N-terminal threonine of β5 (Fig. 5*A*) and inhibits CP catalytic activity, with weaker activity toward β1 and β2 (105). Proteasome inhibition by bortezomib promotes IκB accumulation, suppresses NF-κB signaling, and induces proteotoxic stress, leading to endoplasmic reticulum stress and caspase-dependent apoptosis (106, 107). Clinical use is limited by resistance and dose-dependent toxicities (108, 109). Carfilzomib, a second-generation inhibitor, contains an epoxyketone pharmacophore that irreversibly modifies the active-site threonine through covalent bond formation (Fig. 5*B*) (49, 110). Compared with bortezomib, it exhibits greater selectivity and binding affinity for β5 (111, 112). Ixazomib is an orally bioavailable, reversible inhibitor that primarily targets β5 and induces apoptosis through proteotoxic stress (113).

These inhibitors preferentially target the chymotrypsin-like activity of β5, consistent with its dominant contribution to bulk proteolysis and the structural features of its active-site pocket (114). Proteasome inhibitors suppress both ubiquitin-dependent and ubiquitin-independent degradation pathways. Their clinical use is associated with hematologic toxicities and peripheral neuropathy, and efficacy in most solid tumors remains limited (115). Marizomib (NPI-0052) irreversibly inhibits β1 (IC_50_ 430 nM), β2 (IC_50_ 28 nM), and β5 (IC_50_ 3.5 nM) and has shown activity in clinical studies of MM (116, 117, 118, 119). Together, structural and pharmacologic studies establish that differential architecture of the β-subunit active sites provides a mechanistic foundation for subunit-selective inhibition. While β5-directed inhibitors have achieved substantial clinical success, broader targeting of multiple catalytic sites may offer complementary therapeutic potential but at the cost of increased toxicity. Rational design guided by detailed structural understanding will therefore be critical for advancing proteasome-targeted therapies.

Intrinsically disordered proteins accumulate abnormally in neurodegenerative diseases such as Alzheimer’s and Parkinson’s diseases, thereby disrupting protein homeostasis (120, 121, 122). Recent studies suggest that engineering hyperactive 20S proteasome (α3ΔN) can efficiently clear intrinsically disordered proteins and misfolded proteins, reduce oxidative damage, and improve proteostasis in *C. elegans*, suggesting potential therapeutic relevance for neurodegenerative diseases (123).

Based on structural analyses of the CP (124), two principal approaches have been proposed for the design of 20S activators: one targets the β-subunits to increase catalytic activity, and the other targets the α-subunits to regulate gate opening. Modulation of CP gate opening is more commonly pursued than direct enhancement of β1, β2, and β5 catalytic activity using small molecules. Certain small molecules enhance CP’s peptidase activity by binding to the α-ring and promoting gate opening, although the precise mechanisms remain unclear (125, 126). The RP and other regulatory complexes utilize the HbYX motif to activate the CP by engaging the α-pocket, thereby inducing conformational changes in the α-ring that result in gate opening (59, 127, 128, 129). This observation suggests that HbYX motif-based peptides may have therapeutic potential. A compound termed ZYA, a HbYX motif peptide mimetic, has been reported to activate the mammalian proteasome (130). Molecular docking studies suggest that it could target the α-pocket between α5 and α6 in the human 20S proteasome, analogous to the HbYX motif (130). Furthermore, cryo-EM structures of the ZYA-bound *Thermoplasma acidophilum* 20S proteasome complex revealed that ZYA binds to the 20S intersubunit α-pockets, triggering rearrangement of Lys66 and the pocket back-loop, both of which are involved in proteasome gate opening (131).

## Proteolysis-targeting strategies

**Figure 6:**
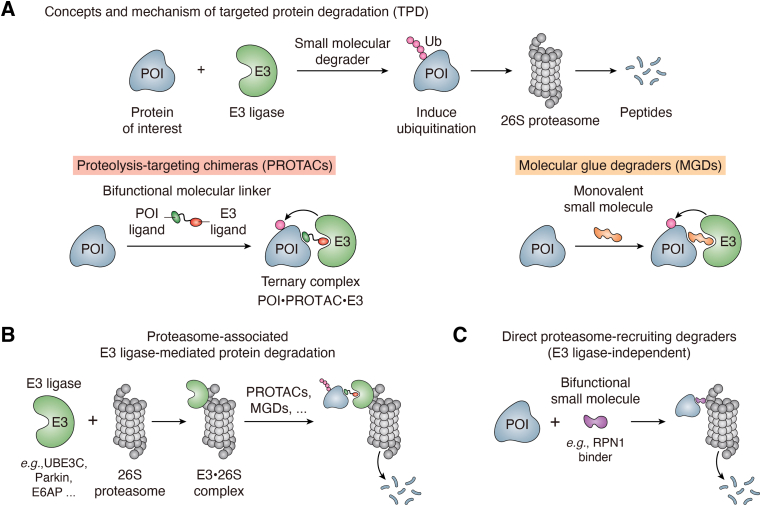
**Schematic illustration of diverse targeted protein degradation (TPD) strategies.***A*, canonical mechanisms of targeted protein degradation. Small-molecule degraders induce proximity between a target protein of interest (POI) and an E3 ubiquitin ligase, promoting polyubiquitination of the POI and its subsequent degradation by the 26S proteasome. This is primarily mediated by two classes of molecules: Proteolysis-targeting chimeras (PROTACs), which are bifunctional molecules containing a POI-binding ligand and an E3 ligand connected by a linker to form a ternary complex; and molecular glue degraders (MGDs), which are monovalent small molecules that induce or stabilize a *de novo* interaction interface between the POI and the E3 ligase. *B*, proteasome-associated E3 ligase-mediated degradation. The E3 ligases (*e.g.*, UBE3C, Parkin, E6AP) interact with the 26S proteasome. Degraders that recruit these specific ligases facilitate the formation of an E3-26S complex, enabling highly efficient, localized delivery of the ubiquitinated substrates to the proteasome. *C*, direct proteasome-recruiting degraders (E3 ligase-independent approach). This alternative strategy employs small bifunctional molecules to directly tether the target protein to specific proteasomal receptors. This approach bypasses the requirement for E3 ubiquitin ligases, directly recruiting the substrate to the 26S proteasome for proteolytic cleavage into peptides. POI, protein of interest; Ub, ubiquitin.

Targeted protein degradation (TPD) is a therapeutic strategy aimed at selectively eliminating disease-associated proteins (132). Unlike conventional inhibitors, TPD functions through a catalytic, substoichiometric mechanism in which a single degrader molecule can induce the degradation of multiple target proteins, thereby enabling pharmacologically meaningful effects without sustained target occupancy and helping to overcome traditionally “undruggable” targets (133, 134). This approach primarily relies on small-molecule degraders that induce proximity between a target protein and an E3 ubiquitin ligase, resulting in substrate polyubiquitination and subsequent proteasomal degradation (135, 136, 137, 138). These degraders are broadly classified into proteolysis-targeting chimeras (PROTACs) and molecular glue degraders (MGDs). PROTACs are bifunctional molecules composed of two ligands connected by a linker: one binds the target protein, while the other engages an E3 ligase, thereby recruiting the substrate to the ligase surface and promoting its ubiquitylation and degradation (139, 140). In contrast, molecular glues are typically monovalent small molecules that induce or stabilize a novel protein–protein interaction interface between an E3 ligase and a neosubstrate, facilitating selective degradation through a distinct but conceptually related mechanism (Fig. 6*A*) (141, 142).

Notably, certain E3 ligases, including UBE3C, E6AP, and Parkin, have been reported to associate with the RP (29, 65, 143). Accordingly, PROTACs or MGDs that recruit such proteasome-associated E3 ligases may enable efficient and direct substrate delivery to the 26S proteasome, thereby enhancing degradation specificity and efficiency (Fig. 6*B*). Although traditional PROTACs and MGDs do not directly modulate proteasome catalytic activity, recent structural studies of UBE3C-retrofitted proteasome suggest the possibility of a UBE3C-targeted PROTAC or MGD may be more effective in delivering neosubstrate to the proteasome for enhanced degradation and thus highlight an alternative paradigm for proteasome-based drug discovery.

Beyond canonical E3 ligase–mediated ubiquitylation, certain shuttle factors can directly deliver substrates to the 26S proteasome (21). Building on this concept, direct proteasome-recruiting degraders have been developed to tether target proteins to proteasomal subunits, thereby bypassing specific E3 ligase dependency (Fig. 6*C*). For example, a new bifunctional small molecule was found to simultaneously engage RPN1 and BRD4, enabling substrate degradation through direct proteasome recruitment (144). Structure-guided approaches may exploit ligands that engage proteasomal receptors such as RPN1 or RPN13, or proteasome-associated shuttle factors, to promote substrate recruitment and entry into the degradation pathway (32). This approach holds promise for overcoming resistance arising from mutations in specific E3 ligases and may significantly expand the repertoire of degradable proteins.

## Outlook

High-resolution cryo-EM analyses have resolved numerous substrate-processing states of the 26S proteasome under various conditions. Collectively, these advancements reveal not only ATPase-driven conformational transitions that coordinate ubiquitin recognition, deubiquitylation, unfolding, and CP gate opening, but also how the proteasome is regulated by the DUBs, E3 ligase and other cellular cofactors. Despite the expansion of the structural “universe” of the proteasome, the existing cryo-EM reconstructions of the proteasomes are only the tip of the iceberg, presumably representing the most readily observable conformers. It remains to be seen if and how the proteasomes in cells interact with many other cofactors or other complex machinery in essential functions ranging from protein quality control to precision control of protein turnover.

The expanding knowledge on the structural landscape of the 26S proteasome across functional states is also enabling structure-guided and computation-assisted modulation of both catalytic and regulatory components. TPD technologies further exploit the UPS. The efficiency of degrader-induced turnover depends not only on ternary complex formation and ubiquitin chain topology but also on proteasome recruitment and substrate-processing kinetics. Interactions between E3 ligases and proteasome subunits, as well as proteasome-associated shuttle factors, suggest potential mechanisms for more direct coupling between ubiquitylation and degradation. Future structural studies will help expand our current understanding of E3–proteasome interaction beyond UBE3C/Hul5. It remains to be seen if a more generic mechanism exists to allow more E3 ligases to directly interact with the proteasome.

Proteasome-mediated proteolysis is governed by coordinated catalytic activity and dynamic communication between the CP and RP. In cancer cells, elevated proteotoxic burden creates a heightened dependence on proteasomal degradation, rendering this system a biochemical vulnerability. Inhibition of the proteasome disrupts regulated proteolytic flux and induces proteotoxic stress that preferentially impairs malignant cell survival. Clinically approved CP inhibitors preferentially target the β5 catalytic subunit, forming covalent adducts with the N-terminal Thr1 and thereby occluding the substrate-binding site within the catalytic chamber. Structural and enzymological studies have clarified modes of inhibitor engagement. However, the conformational consequences of subunit-selective inhibition across distinct functional states of the 26S proteasome remain incompletely understood (145). On the other hand, synthetic activators of the proteasome offer a viable strategy to enhance degradation of therapeutic targets and could be used in combination with PROTACs or MGDs toward traditionally undruggable proteins. By decoding how the UPS is precisely regulated, more protein-interaction cofactors could be exploited as potential therapeutic targets *via* rational *de novo* design of intervention molecules, thus opening new opportunities for addressing unmet medical needs beyond PROTACs or MGDs.

## Conflict of interest

The authors declare that they have no conflicts of interest with the contents of this article.
